# Effect of chest physiotherapy on cystic fibrosis sputum nanostructure: an experimental and theoretical approach

**DOI:** 10.1007/s13346-022-01131-8

**Published:** 2022-03-14

**Authors:** Michela Abrami, Massimo Maschio, Massimo Conese, Marco Confalonieri, Francesco Salton, Fabio Gerin, Barbara Dapas, Rossella Farra, Alessandra Adrover, Gesmi Milcovich, Claudia Fornasier, Alice Biasin, Mario Grassi, Gabriele Grassi

**Affiliations:** 1 Department of Engineering and Architecture, University of Trieste, Via Valerio 6/A, 34127 Trieste, Italy; 2 Institute for Maternal and Child Health, IRCCS Burlo Garofolo, Via dell Istria, 65, 34137 Trieste, Italy; 3 Department of Medical and Surgical Sciences, Foggia University, Ospedali Riuniti, Via L. Pinto, 1, 71122 Foggia, Italy; 4 Pulmonology Department, Cattinara University Hospital, Strada di Fiume 447, 34149 Trieste, Italy; 5 Department of Life Sciences, Cattinara University Hospital, Trieste University, Strada di Fiume 447, 34149 Trieste, Italy; 6 Clinical Department of Medical, Surgical and Health Sciences, Cattinara University Hospital, Trieste University, Strada di Fiume 447, 34149 Trieste, Italy; 7 Department of Chemical Engineering, Materials and Environment, Sapienza University of Roma, Via Eudossiana 18, 00184 Rome, Italy; 8 School of Chemical Sciences, Dublin City University (DCU), Dublin, Ireland

**Keywords:** Cystic fibrosis, Sputum, Rheology, Low field NMR, Mesh size distribution, Drug delivery

## Abstract

Cystic fibrosis (CF) is a disease characterized by the production of viscous mucoid secretions in multiple organs, particularly the airways. The pathological increase of proteins, mucin and biological polymers determines their arrangement into a three-dimensional polymeric network, affecting the whole mucus and impairing the muco-ciliary clearance which promotes inflammation and bacterial infection. Thus, to improve the efficacy of the drugs usually applied in CF therapy (e.g., mucolytics, anti-inflammatory and antibiotics), an in-depth understanding of the mucus nanostructure is of utmost importance. Drug diffusivity inside a gel-like system depends on the ratio between the diffusing drug molecule radius and the mesh size of the network. Based on our previous findings, we propose the combined use of rheology and low field NMR to study the mesh size distribution of the sputum from CF patients. Specifically, we herein explore the effects of chest physiotherapy on CF sputum characteristic as evaluated by rheology, low field NMR and the drug penetration through the mucus via mathematical simulation. These data show that chest physiotherapy has beneficial effects on patients, as it favourably modifies sputum and enhances drug penetration through the respiratory mucus.

**Graphical abstract:**

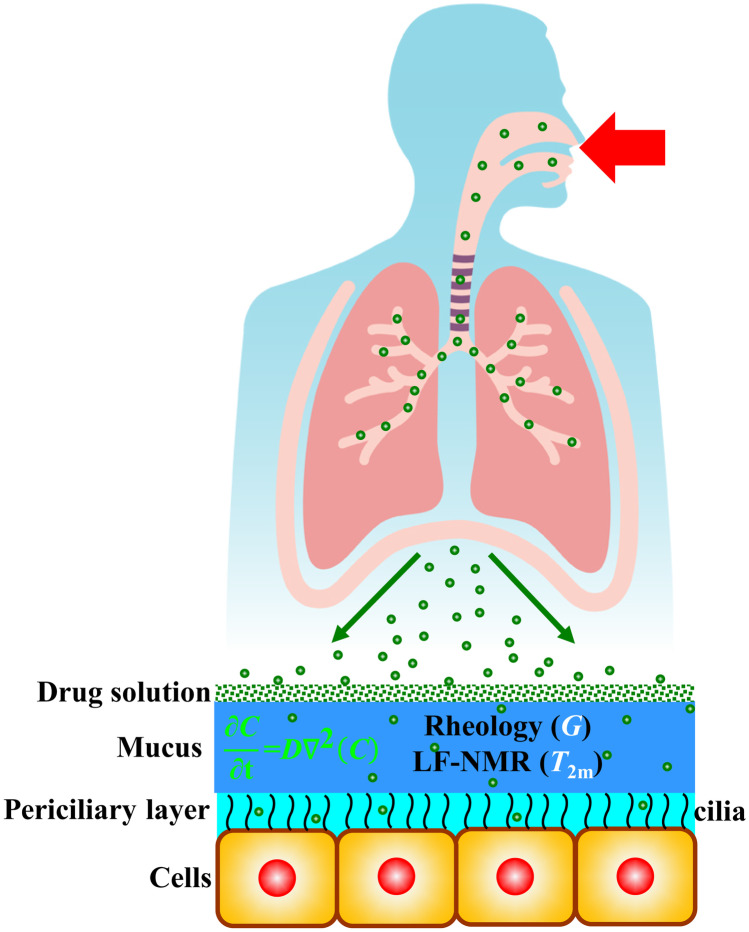

## Introduction

Cystic fibrosis (CF) is one of the most common lethal genetic diseases in people of Caucasian origin [1, 2]. The disease is caused by mutations in the gene encoding the cystic fibrosis transmembrane conductance regulator (CFTR). The CFTR gene encodes an *ATP*-regulated chloride channel present within the apical surface of epithelial cells. Dis-functional CFTR determines a decreased surface liquid volume and increased mucus viscosity in many organs, mainly to the airway. In the healthy lungs, inhaled particles are transported out of the lung by cilia movement, within a process called muco-ciliary clearance. The increased viscosity of mucus in CF patients impairs muco-ciliary clearance determining mucus stagnation, thus favouring bacterial lung infections, a typical event in CF patients [2]. In this regard, CF patients are often colonized by *S. aureus* in the early childhood [3]. *S. aureus* is replaced during disease progression by *P. aeruginosa* as the dominant pathogen. By the time of reaching adulthood, up to 80% of CF patients are chronically infected by *P. aeruginosa*. These chronic pulmonary infections are associated with a rapid decline in lung functions, high morbidity and short life expectancy [4]. One of the most striking hallmarks of *P. aeruginosa* in chronically infected CF patients is the conversion to a mucoid phenotype of the mucus due to the increased production of alginates [5–7], natural polymers composed of mannuronic and guluronic acid units arranged in a block-wise pattern [8]. Alginates overproduction can contribute to a strong inflammatory response sustained by immune cells that can influence lung epithelial cells [9–11]. During inflammation, substances like proteins, mucin and biological polymers increase in mucus as well as the increased alginate production. These compounds organize into a three-dimensional polymeric network, affecting the whole mucus, impairing the muco-ciliary clearance, promoting bacterial infection and hindering drug diffusion to the epithelial cells.

The nanostructure of the network requires an in-depth understanding, in order to improve the efficacy of the drugs usually employed in CF therapy (e.g., mucolytics, anti-inflammatory and antibiotics) when administered alone or in combination with chest physiotherapy. Indeed, drug diffusivity inside a gel-like system depends on the ratio between the diffusing drug molecule radius and the network mesh size. This parameter has driven several research studies focus to date, due to its relevance on the CF sputum/mucus properties [12]. Hanes et al. demonstrated that sputum/mucus is a heterogeneous medium made up of mainly physical cross-linked polymeric regions, embedded in a low viscosity, water-like and fluid [12–14]. According to their complex structure, both the macro- and the micro-rheological mucus/sputum properties have been studied by classical strain/stress controlled rheometers and an innovative particle tracking approach [13]. The network mesh size was determined relaying on rheological sputum characteristics [14] and probe particles tracking [15]. However, as clearly pointed out by Bhat and co-workers [16–18], drug diffusion through mucus can be also affected by drug binding to the glycoproteins. In addition, drug action can be reduced by non-physiological ionic strength, pH, mucociliary clearance and biochemical alterations of mucus [18].

Based on our previous findings, we herein propose the synergic combination of rheology and low-field nuclear magnetic resonance (*LF-NMR*) to study the mesh size distribution changes of mucus in CF patients (obtained by voluntary expectoration and from now on called “sputum”), prior and after chest physiotherapy. Indeed, while *LF-NMR* can provide for info about sputum structure, depending on the magnetic relaxation of water hydrogen trapped in the network, rheology can yield to further insights on the nanostructure, relaying on the mechanical relaxation of the polymeric network chains. Furthermore, *LF-NMR* allows determining the spin–spin relaxation time (*T*_2m_) of the water hydrogens, whereas rheology allows measuring the shear modulus *G* of the sputum. Remarkably, it is possible to determine important features of the sputum nanostructure and further develop innovative drug penetration range, on the *T*_2m_ and *G* knowledge. In particular, the present work focuses on the effects that chest physiotherapy has on sputum characteristic leant on rheology/*LF-NMR* analysis. Therefore, we hypothesize that chest physiotherapy can also influence the sputum nanostructure, representing a pivotal information for clinicians in order to optimize the therapy, for future precision and personalized medicine approach.

## Materials and methods

### Samples collection

Sputum samples were provided by the Burlo Garofolo Hospital, following a procedure approved by the Ethics Committee (prot n. 496/2916, CI M-11, 22–3-2016). Written informed consent was obtained from each patient. One sputum specimen was collected before and after chest physiotherapy session in randomly selected adult and young patients able to expectorate. Non-expectorant patients were excluded, as were subjects ≤ 7 years of age. The chest physiotherapy technique is set up on patient to patient even if the technique most used for the patients enrolled in this study is the pep-mask. Sometimes, autogenous drainage has also been used. Chest physiotherapy was provided as standard procedure for CF patients, to improve clearance, both by using positive expiratory pressure devices and standardised physiotherapist-guided chest applied manoeuvres, as well as effective breathing exercise [19].

The aforementioned criteria allowed for the recruitment of most patients who attended Burlo Garofolo Hospital for a clinical visit during our study period (from January 2019 to December 2020). Spontaneously expectorated (1–2 mL) sputum was collected from *CF* patients in sterile cups and immediately used for *T*_2m_ determination. The samples were then transferred from the *LF-NMR* glass tube to a rheometer device for analysis. Patient’s lung functionality, performed after chest physiotherapy, was evaluated by the Burlo Garofolo Hospital by means of *FEV*_1_ (forced expiratory volume in the first second), i.e. the volume of air that can be forced out in one second after taking a deep breath.

### Rheology

The viscoelastic properties of the analysed samples were determined by means of two rheological tests called, respectively, *stress sweep* and f*requency sweep* test. These experiments require the application of a sinusoidal stress ($\tau$) to the sample in order to measure the related oscillatory deformation ($\gamma$). *Stress sweep* tests were lead applying a sinusoidal stress of constant frequency (*f* = 1 Hz) and increasing amplitude to detect the limit of the linear viscoelastic range. *Frequency sweep* tests, performed in the linear viscoelastic range, as to prevent any sample structure damaging, were lead in the frequency range 10–0.01 Hz. The output of the frequency sweep tests, i.e. the elastic (*G*’) and viscous (*G*’’) moduli dependence on pulsation $\omega$ (= 2$\pi$*f*) (mechanical spectrum), were fitted by the generalized Maxwell model [20]:

1 $${\text{G}}^{^{\prime}} \, \text{=} \, \sum\nolimits_{\text{i=1}}^{{\text{n}}_{\text{R}}}{\text{g}}_{\text{i}}\frac{{\left({\lambda }_{\text{i}}\omega \right)}^{2}}{\text{1} + {\left({\lambda }_{\text{i}}\omega \right)}^{2}}$$

2 $${\text{G}}^{^{\prime\prime}} \, \text{=} \, \sum\nolimits_{\text{i=1}}^{{\text{n}}_{\text{R}}}{\text{g}}_{\text{i}}\frac{\left({\lambda }_{\text{i}}\omega \right)}{\text{1} + {\left({\lambda }_{\text{i}}\omega \right)}^{2}}$$

where *n*_R_ is the number of Maxwell elements considered, whereas *g*_*i*_ (ith elastic constant), $\eta$_i_ (ith viscosity) and $\lambda$_i_ (ith relaxation time) represent model fitting parameters. Model fitting was performed assuming that $\lambda$_i_ were scaled by a factor 10 ($\lambda$_i+1_ = 10 $\lambda$_i_) [20]. Although other fitting strategies could have been followed, this approach enables getting a wider and, thus, clearer, relaxation spectrum (*g*_i_ *vs* $\lambda$_i_). The *n*_R_ determination was performed according to a statistical procedure, in order to minimize the product N*$\chi$^2^, where *N* indicates the number of fitting parameters and $\chi$^2^ is the sum of the squared errors [21]). The sample shear modulus *G* was evaluated as the sum of all *g*_*i*_ (*G* = $\Sigma$*g*_i_) [22]. Therefore, Flory theory [23] allows to evaluate the polymeric network crosslink density, $\rho$_x_, defined as the moles of junction points between different polymeric chains per hydrogel unit volume, based on previous *G* determination:

3 $${\rho }_{\text{x}}={\text{G}}/{\text{RT}}$$

where *R* is the universal gas constant and *T* is the absolute temperature. Therefore, the equivalent network theory [24] enables the determination of the average mesh size $\xi$_a_:

4 $${\xi }_{\text{a}} =\sqrt[{3}]{{6}/\pi {\rho }_{\text{x}}{{\text{N}}}_{\text{A}}}$$

where *N*_A_ is the Avogadro number.

### Low-field NMR

NMR is based on hydrogen atoms dipole reaction to the external constant magnetic field (*B*_0_) switch where they are embedded. Indeed, inside *B*_0_, hydrogen atoms dipoles tend to line up in the *B*_0_ direction so that, globally, they give origin to the induced magnetization vector *M*, oriented in the *B*_0_ direction. Due to *B*_0_ perturbation, realized by the application of a proper radio frequency pulse *B*_1_ perpendicular to *B*_0_, *M* rotates in the *XY* plane of *B*_1_ (that perpendicular to *B*_0_). Upon *B*_1_ removal, *M* tends to line up again to the *B*_0_ direction (relaxation) so that its *XY* component (*M*_XY_) diminishes with time and its component in the *B*_0_ direction (conventionally the vertical or *Z* one; *M*_Z_) increases in time. The mathematical description of the relaxation process (i.e. the *M*_XY_ time reduction) is given by the solution of the magnetization diffusion equation proposed by Brownstein and Tarr [25, 26]:

5 $${\text{I}}({\text{t}}) ={\sum }_{\text{i=1}}^{\text{m}}{{\text{A}}}_{\text{i}}{{\text{e}}}^{(-{\text{t}}/{\text{T}}_{\text{2i}})}$$

where *t* is time, *I*(*t*) is the ratio between the time dependent value of *M*_XY_ and its maximum value (*M*_XYmax_) occurring just after *B*_1_ removal, *T*_2i_ represent the ith spin–spin or transverse relaxation times, while *A*_i_ are “weights” proportional to the number of hydrogen atoms whose dipoles relaxation is characterized by *T*_2i_. Equation (5) simply states that the relaxation process is the result of “*m*” exponential relaxation processes each one characterized by its own relaxation time (*T*_2i_) and weight (*A*_i_). The determination of the unknown couples (*T*_2i_, *A*_i_) was performed by Eq. (5) fitting to the experimental *I*(*t*) values (*I*_s_(*t*)), and the number, *m*, of exponential decays appearing in Eq. (5) was determined per the statistics applied in the Rheology section [21].

The average relaxation time (*T*_2m_) and the average inverse relaxation time ((1/*T*_2_)_m_), depending on several variables such as temperature, *B*_0_ strength, and the presence of a disperse phase in the system as it happens in hydrogel system [26], can be defined by:

6 $${\text{T}}_{\text{2m}}={\sum }_{\text{i=1}}^{\text{m}}{{\text{A}}}_{\text{i}}{{\text{T}}}_{\text{2i}}/{\sum }_{\text{i=1}}^{\text{m}}{{\text{A}}}_{\text{i}} \qquad {\left(\frac{1}{{\text{T}}_{2}}\right)}_{\text{m}}={\sum }_{\text{i=1}}^{\text{m}}\frac{{\text{A}}_{\text{i}}}{{\text{T}}_{\text{2i}}}/{\sum }_{\text{i=1}}^{\text{m}}{{\text{A}}}_{\text{i}}\qquad{{\text{A}}}_{\text{i}\%}={100}{\text{A}}_{\text{i}}/{\sum }_{\text{i=1}}^{\text{m}}{{\text{A}}}_{\text{i}}$$

when *T*_2m_ is evaluated as the inverse of (1/*T*_2_)_m_, it is named *T*_2mb_.

While Eq. (5) provides the discrete relaxation time distribution represented by the *m* couples (*A*_i%_-*T*_2i_), it is possible to determine the continuous distribution according to what suggested by Whittal and MacKay [27]:

7 $${\text{I}}\left({\text{t}}\right)=\int_{T_{2\text{min}}}^{T_{2\text{max}}}{\text{a}}\text{(}{\text{T}}_{2}\text{)}{\text{ exp}}\left\{-\frac{\text{t}}{{\text{T}}_{2}}\right\}{\text{d}}{\text{T}}_{2}$$

where *T*_2max_ (= 10^4^ ms) and *T*_2min_ (= 0.1 ms) indicate, respectively, the lower and upper values of the continuous *T*_2_ distribution, *a*(*T*_2_) is the unknown amplitude of the spectral component at the relaxation time *T*_2_, while exp{-*t*/*T*_2_} represents the decay term. Equation (7) represents the “continuous” expression of Eq. (5). This means that while Eq. (5) describes the *M*_XY_ relaxation process by means of a limited (discrete) number of initially unknowns *T*_2i_, Eq. (7) makes use of a much higher number of known relaxation times belonging to the interval (*T*_2min_ – *T*_2max_) and comprehending all the possible relaxation times characterizing the sample under study (continuous distribution of relaxation times).

In order to fit the experimental *M*_XY_ time decay (*I*_s_(*t*)) by Eq. (7) and get the continuous *T*_2_ distribution (the unknowns *A*_i_ = *a*_i_(*T*_2i_)*$\Delta$*T*_2i_), the following discretization was applied [27]:

8 $${\text{I}}\left({\text{t}}\right)\approx \sum\nolimits_{\text{i=1}}^{\text{N}}{{\text{a}}}_{\text{i}}{{\text{e}}}^{\left\{-\frac{\text{t}}{{\text{T}}_{\text{2i}}}\right\}}\left({\text{T}}_{\text{2i+1}}-{\text{T}}_{\text{2i}}\right)\text{=}\sum\nolimits_{\text{i=1}}^{\text{N}}{{\text{A}}}_{\text{i}}{{\text{e}}}^{\left\{-\frac{\text{t}}{{\text{T}}_{\text{2i}}}\right\}}$$

where the range of the *T*_2_ distribution (*T*_2min_ – *T*_2max_) was logarithmically subdivided into *N* = 200 parts (higher *N* values were unnecessary). Because of the noise disturbing the measure of *I*_s_, the fitting procedure must not minimize the $\chi$^2^ statistic, but a smoothed definition of it (${\chi }_{\text{s}}^{2}$) [27]:

9 $${\chi}_{\text{s}}^{2}=\sum\nolimits_{\text{i=1}}^{\text{N}}{\left(\frac{{\text{I}}_{\text{s}}\left({\text{t}}_{\text{i}}\right)-{\text{I}}\left({\text{t}}_{\text{i}}\right)}{{\sigma }_{\text{i}}}\right)}^{2}+\mu \sum\nolimits_{\text{i=1}}^{{\text{N}}-2}{\left|{\text{A}}_{\text{i+2}}-{{2}{\text{A}}}_{\text{i+1}}+{\text{A}}_{\text{i}}\right|}^{2}$$

where $\sigma$_i_ is the i^th^ datum standard deviation and $\mu$ is the weight of the smoothing term (second summation in Eq. (9)) proposed by Provencher [28]. Although different criteria can be used to determine $\mu$, the strategy proposed by Wang [29] was applied. Based on this strategy, the correct $\mu$ value is that occurring just below the heel (slope variation) of the function ln($\chi$_s_) vs ln($\mu$). In the present work, $\mu$ = 150 was determined.

The discrete and continuous *T*_2_ distribution can be transformed into hydrogel mesh size distribution resorting to one of the fundamental relations of the low-field NMR field. This relation, based on the solution of the magnetization diffusion equation proposed by Brownstein and Tarr [25, 26], establishes the link between (1/*T*_2_)_m_ and the ratio of the surface (*S*) of the dispersed/solubilized substances in the sample and the volume (*V*) of the sample water molecules:

10 $${\left(\frac{1}{{\text{T}}_{2}}\right)}_{\text{m}}=\frac{1}{{\text{T}}_{\text{2H2O}}}+\frac{\text{S}}{{\text{V}}}{\text{M}}$$

where *T*_2H2O_ is the bulk protons relaxation time (i.e. the water proton relaxation time in the absence of polymer, the so-called free water relaxation time ≈ 3700 ms at 37 °C and *B*_0_ = 0.47 T [30]) and M (length/time) is a physical parameter, named relaxivity, which represents the effect of the surface of polymer chains on water proton relaxation. Indeed, M is equal to the ratio between thickness and relaxation time of the bound water layer adhering to the solid surface. Equation (10), stating that (1/*T*_2_)_m_ depends on (*S*/*V*), clearly establishes the relation between the relaxation time and the spatial organization of the sample network that heavily affects the *S*/*V* ratio [26]. For instance, in many polymeric systems, crosslinking induces a spatial reorganization of the polymeric chains contained in the original solution that involves the increase of the ratio *S*/*V* [31, 32]. This, in turn, reflects in the increase of (1/*T*_2_)_m_ and in the decrease of *T*_2m_.

Despite its theoretical importance, Eq. (10) can be re-written in a more useful form, based on the fibre cell [26] and Scherer theories [33]. At this purpose, Abrami et al. [34] demonstrated that, for a hydrogel polymer volume fraction $\phi$ < 0.6, the term (*S*/*V*) can be expressed as a function of the average mesh size $\xi$_a_ of the polymeric network (error < 5%):

11 $$\frac{\text{S}}{{\text{V}}}=\frac{2}{{\xi }_{\text{a}}\sqrt{\frac{{\text{C}}_{0}}{{\text{C}}_{1}}\frac{1-0.58\varphi }{\varphi }}}$$

where *C*_0_ and *C*_1_ are two constants depending on the mesh architecture and equal, respectively, to 1 and 3$\pi$ in the case of cubic mesh [33]. Thus, Eq. (10) becomes

12 $${\left(\frac{1}{{\text{T}}_{2}}\right)}_{\text{m}}=\frac{1}{{\text{T}}_{\text{2H2O}}}+ \text{2} \frac{\text{M}}{{\xi }_{\text{a}}\sqrt{\frac{{\text{C}}_{0}}{{\text{C}}_{1}}\frac{1-0.58\varphi }{\varphi }}}$$

while Eq. (12) refers, averagely, to all the polymeric network meshes, similar expressions can be written for meshes of different dimensions ($\xi$_i_), assuming the M independence on the mesh size [26]:

13 $$\frac{1}{{\text{T}}_{\text{2i}}}=\frac{1}{{\text{T}}_{\text{2H2O}}}+ \text{2} \frac{\text{M}}{{\xi }_{\text{i}}\sqrt{\frac{{\text{C}}_{0}}{{\text{C}}_{1}}\frac{1-0.58\varphi }{\varphi }}}$$

where *T*_2i_ is the relaxation time of water protons trapped in polymer meshes of size $\xi$_i_. The bi-univocal correspondence between *T*_2i_ and $\xi$_i_ only holds in the fast-diffusion regime (typical of gels), i.e. when the mobility of water molecules, expressed by their self-diffusion coefficient *D* (3.04 × 10^−9^ m^2^/s at 37 °C [35]), is large whether compared to the rate of magnetization loss (*R*_c_ × M) (i.e. *R*_c_*M/D ≪ 1). In the slow diffusion regime, relaxation of all the water protons contained in the volume of a mesh of size $\xi$_i_ is not described by just one *T*_2i_ but several *T*_2i_. *R*_c_ indicates the radial distance from the polymer chain axis where the effect of polymeric chains on water proton relaxation becomes negligible. This can be expressed by [26]

14 $${\text{R}}_{\text{c}}=\frac{{\text{r}}_{\text{f}}}{\sqrt{\varphi }}$$

The combination of Eqs. (12) and (13) allows to conclude that the ratio between $\xi$_i_ and its average value, $\xi$_a_, depends exclusively on the relaxation times *T*_2i_ and *T*_2m_ (except for the free water relaxation time *T*_2H2O_):

15 $$\xi ={\xi }_{\text{a}}\frac{\left({\left(\frac{1}{{\text{T}}_{2}}\right)}_{\text{m}}\text{- }\frac{1}{{\text{T}}_{\text{2H2O}}}\right)}{\left(\frac{1}{{\text{T}}_{\text{2i}}}-\frac{1}{{\text{T}}_{\text{2H2O}}}\right)}$$

Thus, Eq. (5) or Eq. (8) fitting to experimental *M*_XY_ decay (*I*_s_(*t*)) allows determining the discrete or continuous relaxation spectrum (*A*_i%_ − *T*_2i_), whereas Eq. (13) provides for the conversion into the discrete or continuous mesh size distribution of the sputum (*A*_i%_ − $\xi$_i_).

LF-NMR measurements were performed at 37 °C by means of a Bruker Minispec mq20 (0.47 T, Germany). The determination of *T*_2m_ was performed according to the CPMG sequence (Carr–Purcell–Meiboom–Gill) [36] {90°[-τ-180°-τ(echo)]n-*T*_R_} with a 8.36 μs wide 90° pulse, *τ* = 250 μs, and *T*_R_ (sequences repetition rate) equal to 10 s. In order to obtain the final *I*_s_ of 2%, the proper *n* was chosen. *m* was determined according to the statistics applied in the Rheology section [21]. Each spin-echo decay, composed by *n* points, was repeated 36 times (number of scans).

### Drug diffusion

**Fig. 1:**
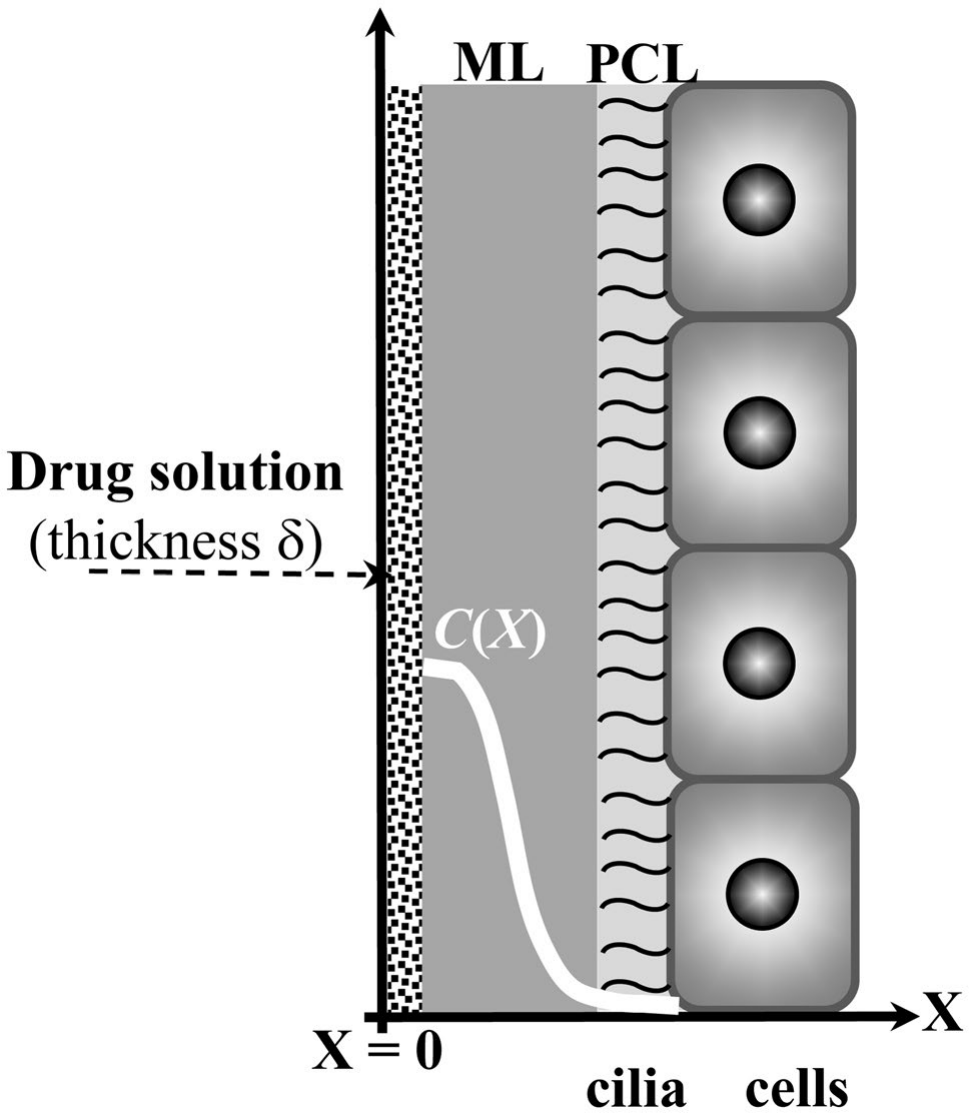
The airway surface liquid (*ASL*) lining the walls of the bronchial tree is composed by the periciliary liquid (*PL*), where cells cilia beat, and by the mucus layer (*ML*). When a drug solution is inhaled, it spreads on the *ML*, and the drug can diffuse inside the *ASL*. White line indicates the hypothetical drug concentration profile inside *ASL*

The walls of the bronchial tree are covered by a very thin layer of a water-like serous fluid, the periciliary liquid (*PCL*), where cells cilia beat with a typical frequency of 20 Hz and amplitude about 5 $\mu$m. On the top of *PCL*, a thin layer of a non-Newtonian viscoelastic fluid lies, named mucus [37] (see Fig. 1).

*PCL* and mucus layer (*ML*) constitute the airway surface liquid (*ASL*) [38]. The viscoelastic nature of mucus depends on the presence of different components such as proteins, alginates, white blood cells, DNA, bacteria and mucin (whose levels are pathologically increased compared with healthy subjects [39]) that lead to a three-dimensional network [40]. Drug permeation inside the *ASL* is a complex phenomenon affected by many factors such as the thickness of *ASL*, which depends on the *ASL* rheological properties and the airflow [37, 41, 42]. Moreover, possible interactions (e.g. electrostatic and hydrogen bonding) between the diffusing drug and the polymeric components of the *ALS* cannot be a priori excluded [43]. Furthermore, as *ASL* is not homogeneous, the drug diffusion coefficient (*D*) should be position dependent [42]. However, assuming that drug transport is mainly affected by diffusion with constant *D*, the prediction of the drug penetration inside *ASL* can be obtained by referring to the one-dimensional Fick’s equation with proper initial and boundary conditions. In detail, it is assumed that at time zero (deposition of drug solution on *ASL*, following inhalation), the drug is not present inside *ASL*, and it is uniformly distributed, at the known concentration *C*_0_, in the solution layer of thickness $\delta$ (see Fig. 1). Boundary conditions involve the existence of an impermeable wall in *X* = 0 (the drug cannot diffuse in the *X* negative direction) and the possibility of drug diffusion in the positive *X* direction up to infinite (therefore, the drug can diffuse inside cells, too). According to these hypotheses, the time evolution of the drug profile concentration (*C*) inside *ASL* is determined by

16 $$\frac{\text{C}}{{\text{C}}_{0}}=\frac{\delta }{\sqrt{{\text{D}}{\text{t}}}\pi }{\text{e}}^{\text{-(}\frac{{\text{X}}^{2}}{{4}{\text{D}}{\text{t}}}\text{)}}$$

where *t* is time. The *D* value to be used in Eq. (15) can be provided by the Lusting-Peppas equation [44] as per Abrami et al. [23]:

17 $${\text{D}} \, = {\text{D}}_{0}(1\text{-}\frac{{2}{\text{r}}_{\text{s}}}{{\xi}_{\text{a}}}){\text{e}}^{-{\text{Y}}(\frac{\varphi}{1-\varphi})}$$

where *r*_s_ is the radius of the drug molecule imagined to be a sphere, *D*_0_ is the drug diffusion coefficient in water, while *Y* is a model parameter that, lacking further information, can be set equal to 1 [44], although it usually ranges between 3 and 30 [45, 46] (see Appendix 1 for some considerations on the potentiality of Eq. (17)). The solid volume fraction φ was set equal to 0.05, this being the most common situation, albeit variability can occur among samples [47]. For what concerns *r*_s_ and *D*_0_, with an attention focus on typical antibiotics used for *FC* patients (ciprofloxacin and tobramycin, for example [48]), reasonable values are 1.1 nm and 3 × 10^−10^ m^2^/s, respectively [49, 50]. Moreover, it is worth mentioning that while the *PCL* thickness ranges between 5 and 10 $\mu$m [51], in healthy subjects and in CF patients, *ML* thickness is similar and, approximately, equal to 25–30 $\mu$m [37, 52]. Vice-versa, patients affected by chronic obstructive pulmonary diseases (COPD) exhibit an *ML* thickness value up to 300 $\mu$m [41].

### Statistical analysis

The nature of the experimental data distribution (normal or not) was evaluated by the Kolmogorov–Smirnov test (KS-test). Based on KS-test results, the Spearman’s correlation coefficient (*r*_sp_) was considered to verify possible direct or inverse correlations among *T*_2m_, *FEV*_1_ and *G*. Correlations among variables were considered significant when *p* <0.05, corresponding to a probability of 95%. Lower probability was associated to a lack of correlation among variables.

## Results and discussion

**Fig. 2:**
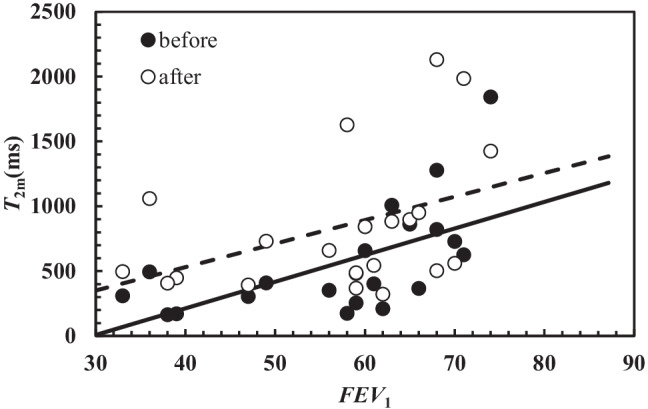
Correlation between *T*_2m_ and *FEV*_1_ before (black circles) and after (white circles) chest physiotherapy. In both cases, the correlation is statistically significant (before: *r*_sp_ = 0.69, *p* = 0.006; after *r*_sp_ = 0.44, *p* = 0.044). The linear relation occurring between *T*_2m_ and *FEV*_1_ is represented by solid line (before: *T*_2m_(ms) = (20.5 ± 6.3) × *FEV*_1_ – (609 ± 366)) and dashed line (after: *T*_2m_(ms) = (18.1 ± 9.0) × *FEV*_1_ – (193 ± 526))

Figure 2 remarkably shows that there is a statistically significant correlation between *T*_2m_ measured in CF sputum and *FEV*_1_ before (dark circles; *r*_sp_ = 0.69, *p* = 0.006) and after (white circles; *r*_sp_ = 0.44, *p* = 0.044) chest physiotherapy. As lung functionality increases with *FEV*_1_ [53], Fig. 2 indicates that *T*_2m_ increase is connected with improved patient clinical conditions. Moreover, the inspection of Fig. 2 reveals that, on average, after chest physiotherapy, *T*_2m_ is higher.

Indeed, the dashed line (connected with the *T*_2m_ vs *FEV*_1_ trend after chest physiotherapy) is characterized by a higher intercept with respect to solid line (connected with the *T*_2m_ vs *FEV*_1_ trend after chest physiotherapy), and the two straight lines share, approximately, the same slope. Thus, the beneficial action of chest physiotherapy is clearly evident.

**Fig. 3:**
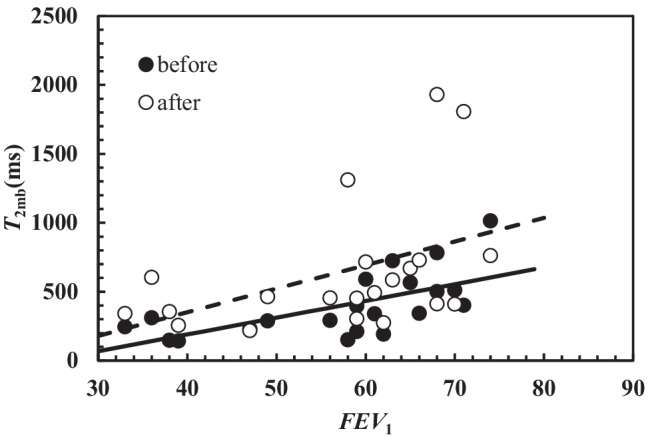
Correlation between *T*_2mb_ and *FEV*_1_ before (dark circles) and after (white circles) chest physiotherapy. In both cases, the correlation is statistically significant (before: *r*_sp_ = 0.73, *p* = 0.0002; after *r*_sp_ = 0.51, *p* = 0.019). The linear relation occurring between *T*_2mb_ and *FEV*_1_ is represented by solid line (before: *T*_2mb_(ms) = (12.2 ± 3.3) × *FEV*_1_ – (295 ± 195)) and dashed line (after: *T*_2mb_(ms) = (17.1 ± 8.0) × *FEV*_1_ – (333 ± 464))

Interestingly, similar correlations between *T*_2mb_ (average relaxation time evaluated as the inverse of (1/*T*_2_)_m_) and *FEV*_1_ have been determined both before and after chest physiotherapy as shown on Fig. 3.

**Fig. 4:**
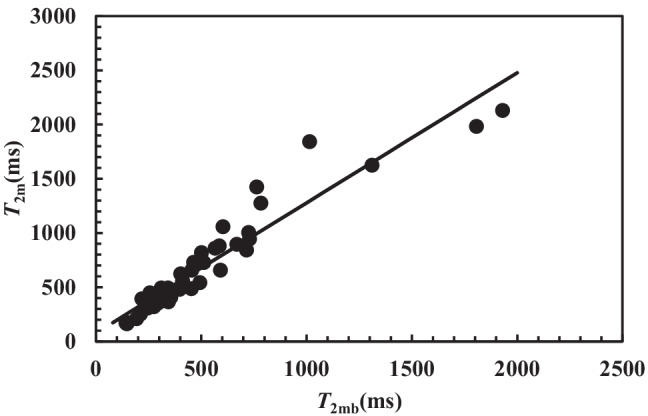
Statistically **s**ignificant correlation between *T*_2m_ and *T*_2mb_ referring to all the 42 samples considered (*r*_sp_ = 0.97, *p* < 0.0001). Solid line represents the linear interpolant: *T*_2m_(ms) = (1.2 ± 0.06) × *T*_2mb_ + (78 ± 41)

This finding is due to the strong correlation existing between *T*_2m_ and *T*_2mb_ (*r*_sp_ = 0.97, *p* <0.0001), as per Fig. 4 for all the analysed samples. Even though considering separately the samples before and after chest physiotherapy, the same strong correlation is demonstrated too.

**Fig. 5:**
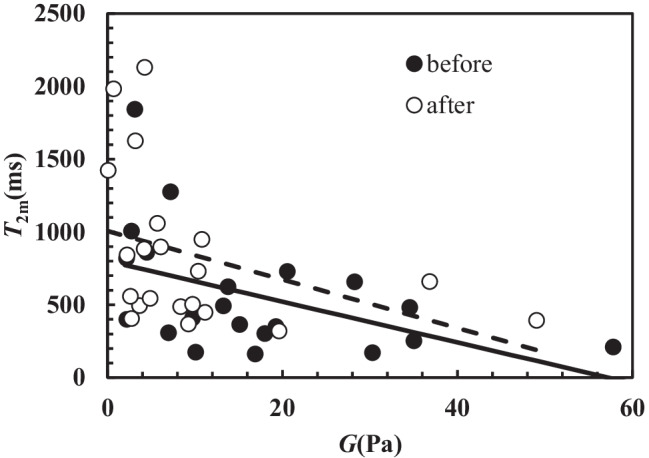
Correlation between *T*_2m_ and *G*. Dark circles indicate samples before chest physiotherapy, while white circles refer to samples after chest physiotherapy. A statistically significant correlation between *T*_2m_ and *G* occurs both before (*r*_sp_ = −0.53, *p* = 0.014; *T*_2m_(ms) = −(14.0 ± 6.0) × *G*(Pa) + (801 ± 131); solid line) and after (*r*_sp_ = −0.495, *p* = 0.023; *T*_2m_(ms) = −(14.6 ± 9.3) × *G*(Pa) + (1006 ± 143); dashed line) chest physiotherapy. The overall correlation is statistically significant (*r*_sp_ = −0.58, *p* = 0.0001; *T*_2m_(ms) = −(16.8 ± 5.2) × *G*(Pa) + (928 ± 97))

**Fig. 6:**
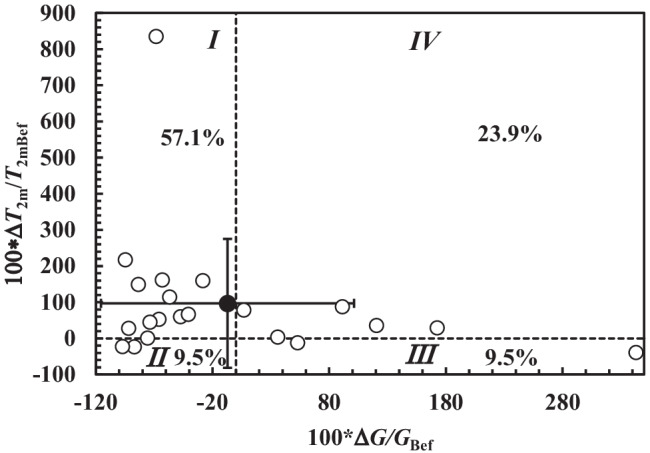
Relative percentage variation of the relaxation time (100*$\Delta$*T*_2m_/*T*_2mBef_) versus the relative percentage variation of the shear modulus (100*$\Delta$*G*/*G*_Bef_) considering samples after and before chest physiotherapy (open circles). Black circle indicates the average value of 100*$\Delta$*T*_2m_/*T*_2mbef_ and 100*$\Delta$*G*/*G*_bef_, while vertical and horizontal solid lines indicate the associated standard deviation in the vertical and horizontal direction

Thus, for what concerns the evaluation of lung functionality in relation to *FEV*_1_, both *T*_2m_ and *T*_2mb_ can be chosen as indicators of patient clinical conditions. Figure 5 shows the significant inverse correlation between *T*_2m_ and the shear modulus *G* on the samples both before and after chest physiotherapy (*r*_sp_ = −0.58, *p* = 0.0001). These evidences support the beneficial effects of chest physiotherapy. Indeed, at fixed *G*, Fig. 5 shows that chest physiotherapy leads to a higher *T*_2m_ (see the solid and dashed lines in Fig. 5). An additional and conclusive proof of chest physiotherapy benefit is displayed in Fig. 6. This picture reports the %, relative (compared to the value before chest physiotherapy, *T*_2mBef_) variation ($\Delta$*T*_2m_) of *T*_2m_ versus the %, relative (with respect to the value before chest physiotherapy, *G*_Bef_) variation ($\Delta$*G*) of *G*. It is possible to detect that in the 57.1% of the cases (section I in Fig. 6), chest physiotherapy leads to both an increase of *T*_2m_ and a decrease of *G*, which indicates the partial breakage of the original mucus polymeric network that yields to a weaker nanostructure. Indeed, the combination of Eqs. (3), (4) and (12) evidences that *T*_2m_ (or *T*_2mb_) is inversely proportional to *G*^1/3^. Therefore, network damaging is due to a *T*_2m_ increase, as per *G* decrease due to a reduction of crosslink density (see Eq. (3)). The direct correlation between *T*_2m_ and *FEV*_1_ evidences that nanostructure variation indicates a better lung functionality. Figure 6, section IV shows that in the 23.9% of the cases, chest physiotherapy determines an increase both in *T*_2m_ and *G*. This behaviour could be explained assuming that the increase of sputum stiffness is not due to the increase of the crosslink density, but to the increase of polymeric fibres strength as the polymeric chains group into thicker fibres (see Fig. 6). This perfectly matches with *G* and *T*_2m_ increase, as *T*_2m_ rises with the decrease of the ratio between the solid (fibres) surface (*S*) in contact with water molecules and the hydrogel water molecule volume (*V*) as per Eq. (10) [22, 54].

**Fig. 7:**
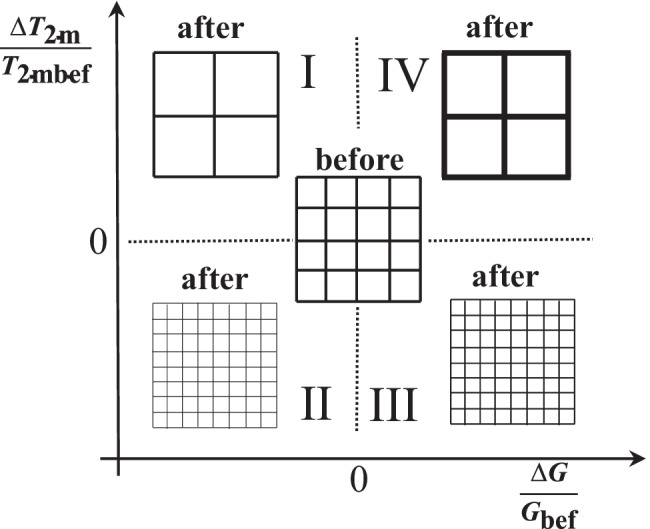
Qualitative effect of chest physiotherapy on sputum nanostructure deduced by the percentage variation of the relaxation time (100*$\Delta$*T*_2m_/*T*_2mbef_) and the relative percentage variation of the shear modulus (100*$\Delta$*G*/*G*_bef_) (sections I–IV are those of Fig. 6). Depending on the relative variation of *T*_2m_ and *G*, different mucus nanostructures can be supposed

Figure 6 section II reveals that in 9.5% of the cases, chest physiotherapy determines both *G* and *T*_2m_ reduction. This proves the increase of the crosslink density, which takes place among thinner and weaker fibres. In the 9.5% of the cases, chest physiotherapy determines a *G* increase and *T*_2m_ decrease (Fig. 6 section III). In this case, *T*_2m_ decrease and *G* increase could be related to mesh size reduction. Overall, the effect of chest physiotherapy on sputum nanostructure is positive, as it determines the average *T*_2m_ increase and the average *G* decrease (see the black dot in section I of Fig. 6). Figure 7 qualitatively sums up the effect of chest physiotherapy on the sputum nanostructure, based on the relative variations of *T*_2m_ and *G* shown in Fig. 6.

**Fig. 8:**
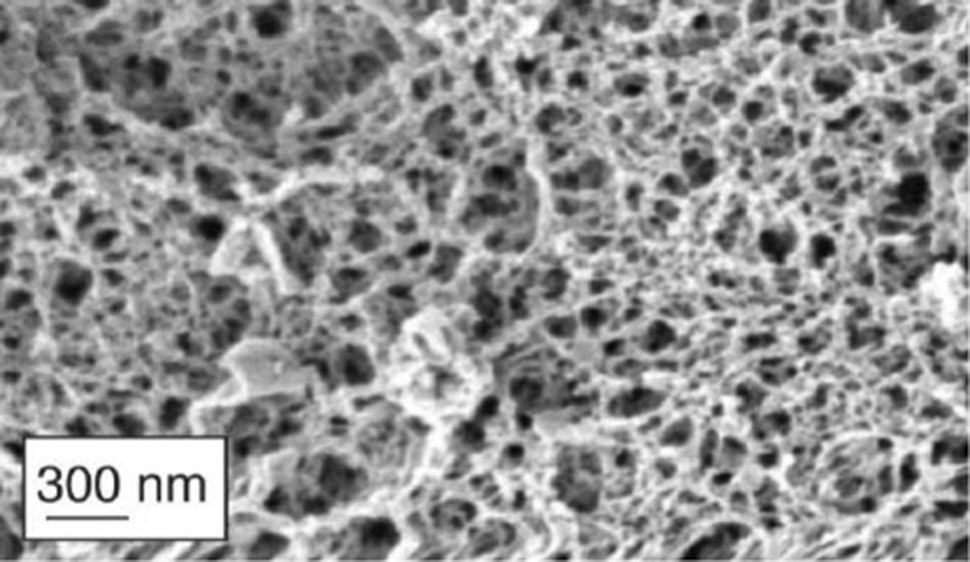
Nanostructure of CF sputum. The complexity of CF nanostructure is increased by the presence of DNA, cell debris and bacteria (not visible in this figure). ( Adapted from Ref [58] with permission from FutureScience)

It is of outmost importance to remind that all the above considerations relay on the interpretation of experimental results in the light of Eqs. (3), (4) and (12). Thus, other experimental techniques (such as TEM/SEM or other image techniques) will be considered. However, the complex nature of CF sputum nanostructure, reported in Fig. 8, and the issues connected to the reliable acquisition and interpretation of images [55–57] make this task absolutely challenging.

Moreover, for what concerns the image analysis, the presence of DNA, cell debris and bacteria can create an extremely complex scenario. On the other hand, this is not a problem for the proposed combined approach (rheology and LF-NMR) as the presence of additional components just alters the *S*/*V* ratio (see Eq. (10)) that reflects in a variation of *T*_2m_. Similarly, the presence of additional components affects the sputum rheological behaviour reflecting in alterations of *G*’ (shall they represent physical or chemical bonds among different polymeric chains) or *G*’’ (shall they stay in the sputum sol fraction). Thus, the presence of DNA, cell debris and bacteria is accounted for by our approach, and it is interpreted as a sort of network architecture modifications.

**Fig. 9:**
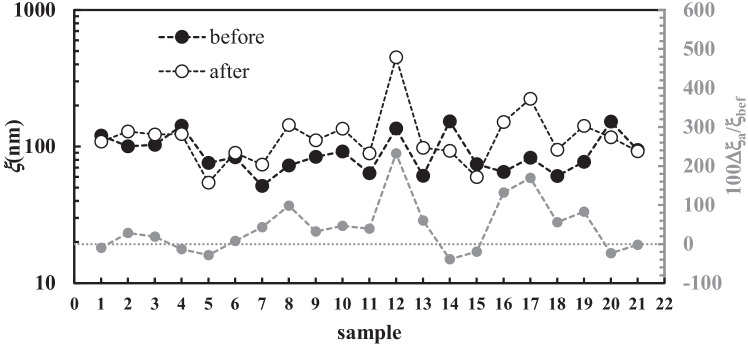
Variation of the average mesh size ($\xi$_a_) for the 21 samples considered (black circles, before chest physiotherapy; white circles after chest physiotherapy). Grey circles indicate the relative percentage variation of the average mesh size $\xi$_a_ (secondary vertical axis)

**Fig. 10:**
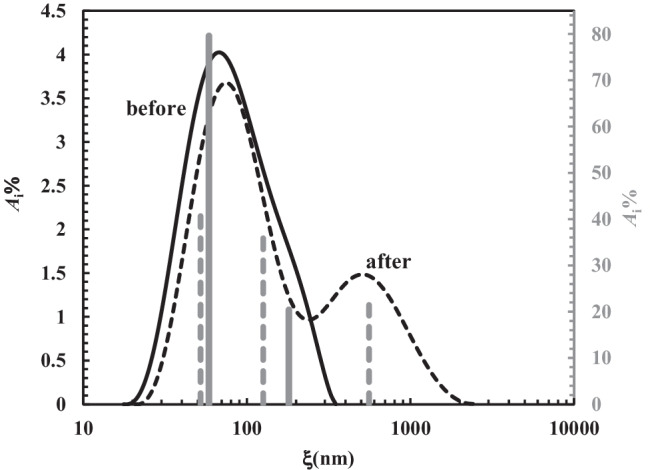
Comparison between the continuous mesh size distribution before (solid black curve) and after (dashed black line) chest physiotherapy referring to samples 11 (see Table 1). Solid grey vertical lines (secondary vertical axis on the right) indicate the discrete mesh size distribution referring the solid black curve. Dashed grey vertical lines (secondary vertical axis on the right) indicate the discrete mesh size distribution referring the dashed black curve

The general soundness of the presented approach is proved by Figs. 9 and 10 showing, respectively, the average mesh size ($\xi$_a_) of all samples and the continuous mesh size distribution referring to sample 11 before and after chest physiotherapy (see Table 1 in Appendix 2). Indeed, according to Hanes and co-workers [58, 59], who applied a sophisticated particles tracking methods, the mesh size distribution of the CF sputum network ranges between 60 and 300 nm, with a mean value of (145 ± 50) nm. Figure 9 shows that, except for one case, $\xi$_a_ refers to the range of 50–250 nm, i.e. within the range indicated by Hanes and co-workers. Moreover, Fig. 10 shows that before chest physiotherapy (unperturbed sputum), the continuous mesh size distribution spans between 20 and 300 nm, perfectly matching Hanes and co-workers findings. Thus, the two different strategies (our bulk rheology-LF-NMR and Hanes’ particle tracking) provide similar results.

Figure 9 also allows to sum up the effect of chest physiotherapy on sputum nanostructure. Indeed, Fig. 9 suggests that, on average, chest physiotherapy leads to an increase of $\xi$_a_, as its relative variation (100$\Delta \xi$_a_/$\xi$_bef_) is mainly positive. Conversely, negative variations are limited in their amplitude (grey circles), with reference to positive ones (see dotted grey line). As per Eqs. (8) and (15), it is possible to determine the variation of the continuous mesh size distribution, based on chest physiotherapy treatment. Figure 10 shows, e.g. this comparison in the case of samples 11 (see Table 1 in Appendix 2).

It is possible to notice that chest physiotherapy pushes towards higher mesh size values ($\xi$) the entire mesh size distribution (compare the black solid line and dashed black line). It also provides for changes in the shape of the distribution, making more evident the second peak, which is just slightly visible before the treatment. This is a pivotal hallmark: the second peak, moving from, about, 200 nm to 500 nm, refers to mesh size range which is typical of healthy sputum.

Moreover, Fig. 10 clearly proves that about 20% of the mesh size distribution is on the normal range size (dashed black line), while, before chest physiotherapy (continuous solid line), 100% of the mesh size refers to the pathological range. Therefore, both before and after chest physiotherapy, the discrete mesh size distribution (grey vertical lines) results in a trend of the continuous one.

**Fig. 11:**
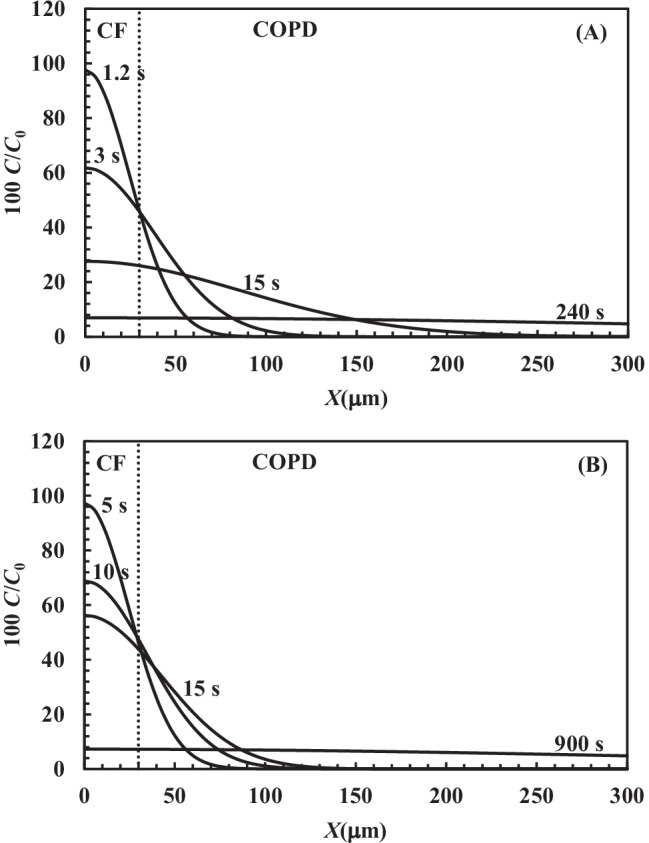
Drug profile concentration (*C*/*C*_0_, solid line) inside *ASL* thickness at different times according to Eq. (16) assuming $\delta$ = 3 $\mu$m. The drug diffusion coefficient has been evaluated according to Eq. (17) assuming *φ* = 0.05, *r*_s_ = 1.1 nm, *D*_0_ = 3 × 10^−10^ m^2^/s and **A** *Y* = 3 (*D* = 2.5 × 10^−10^ m^2^/s) or **B** *Y* = 30 (*D* = 0.6 × 10^−10^ m^2^/s)

Drug delivery through mucus is a complex phenomenon which is on stage for researchers, with a main focus of the current COVID period the world is facing [16–18, 60]. For what concerns drug (e.g. antibiotics, anti-inflammatory and mucolytics) administration by inhalation and in order to achieve this task, detailed information about mesh size are of key importance. Indeed, the entire delivery process implies different steps such as drug deposition on *ASL* (airway surface liquid, sum of the periciliary liquid and mucus layers) and drug diffusion throughout it. Modern strategies ensure that within, about, 7 min, the administered aerosol can get the *ASL* [61, 62]. In the light of the simplifying hypotheses on which Eq. (16) relies, we would like to simulate the kinetics of the drug diffusion process inside *ASL.* At this purpose, the drug diffusion coefficient *D* was evaluated according to Eq. (17) assuming the values of the model parameters (φ = 0.05, *r*_s_ = 1.1 nm, *D*_0_ = 3 × 10^−10^ m^2^/s) presented in the “Diffusion” section. Moreover, the average mesh size was fixed equal to the average value (92 nm) reported in Table 1; *Y* was set equal to 3 or 30 to account for two limiting conditions and $\delta$ = 3 $\mu$m. When *Y* = 3 (this corresponding to a higher drug diffusivity *D* = 2.5 × 10^−10^ m^2^/s), Fig. 11A reveals that within few seconds, the drug can cross the *ASL* typical thickness of healthy subjects and CF patients (≈ 30 $\mu$m).

Indeed, the dimensionless concentration *C*/*C*_0_ achieves about 50% of the initial solution concentration at the *ASL* bottom in a very short time (see the profile concentration curves labelled 1.2 and 3 s in Fig. 11A). For longer times, the *C*/*C*_0_ profile assumes a flatter shape (see the 15 s labelled curve in Fig. 11A) and a uniform distribution is achieved after 240 s through the *ASL* thickness competing to COPD (chronic obstructive pulmonary diseases) patients (300 $\mu$m). COPD has been considered as it is due to an acquired CFTR impairment, resulting in increased lung mucus viscosity similarly to CF. Considering *Y* = 30, this value represents a lower value of the diffusion coefficient (*D* = 0.6 × 10^−10^ m^2^/s). Figure 11B reveals that the diffusion process is slowed down, but *C*/*C*_0_ is about 50% at a penetration depth of 30 $\mu$m after 5 s. Moreover, 900 s are required to get a flat concentration profile up to 300 $\mu$m, i.e. the *ASL* thickness which can be detected in COPD patients. Thus, in the case of CF patients, these simulations indicate that the drug can diffuse on the *ASL* thickness in a few seconds. On the other hand, in the case of COPD patients, 6–15 min are required to get the same effect if *ASL* thickness is about 300 $\mu$m. It is worth to mention that the values of the diffusion coefficients therein reported, assuming *Y* = 3 or 30 (2.5 × 10^−10^ m^2^/s and 0.6 × 10^−10^ m^2^/s, respectively), approximately represent the maximum and minimum *D* range which can be determined by the experimental permeability data through mucus performed by Bhat and co-workers [17, 18] that span from 2.4 × 10^−10^ m^2^/s down to 0.7 × 10^−10^ m^2^/s.

In the case of CF patients, drug diffusion through mucus is not the rate determining step of the whole delivery process. In the worst conditions for COPD patients, drug diffusion can be compared to the inhalation time. However, in both cases, the time required for drug diffusion is short, this witnessing the promising role of inhalation to match pulmonary diseases.

## Conclusions

The characterization of the sputum of CF by the combined use of rheology and *LF*-*NMR* allowed getting information about the nanostructure of sputum derived from the mucus lining the bronchial tree. In particular, this analysis was aimed to determine sputum nanostructure variations, as a result of chest physiotherapy, a routine procedure adopted in CF patients. We could verify that significant nanostructure variations occurred upon chest physiotherapy, leading to beneficial clinical effects for the patients. Interestingly, the combined use of rheology and *LF*-*NMR* allowed to define the nanostructure characteristics that each technique (if used singularly) would have never been able to provide. Moreover, assuming that drugs diffuse inside mucus according to Fick’s law, as well as in the sputum nanostructure, we could determine that in the case of healthy subjects and CF patients, about 7 min from inhalation are needed to get the drug active inside the mucus, i.e. a very short time. In case of COPD patients, this time has been estimated to about 15 min in the worst situation (300 $\mu$m *ASL* thickness). Anyway, in both cases, the time required to get drug action since inhalation resulted to be short. Therefore, the inhalation delivery demonstrated a key role to provide for effective info about pulmonary diseases, such as those afflicting CF and COPD patients.

## Figures and Tables

**Fig. 12:**
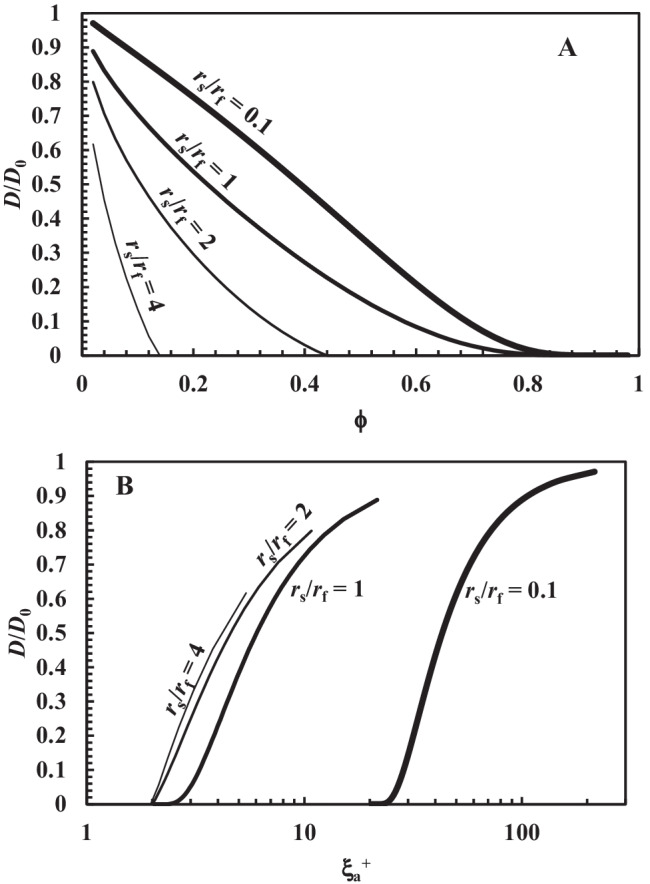
**A** Variation of the ratio *D*/*D*_0_ according to Eq. (18) and **B** Eq. (19) assuming different values of the ratio between the drug molecule radius (*r*_s_) and the radius (*r*_f_) of the fibres constituting the polymeric network pervading the hydrogel. These simulation refer to a cubical mesh organization (*C*_0_ = 1; *C*_1_ = 3$\pi$ [33])

## Data Availability

Where not directly provided in the paper, all the data referring to this paper are available upon request to the corresponding author.

## Appendix 1

The approximation of the Scherer theory [33] by Abrami and co-workers [34] allows to provide a simple relation between the average mesh size $\xi$_a_, the polymer volume fraction φ and the polymeric chains radius *r*_f_:

18 $${\xi }_{\text{a}}={\text{r}}_{\text{f}}\sqrt{\frac{{\text{C}}_{1}}{{\text{C}}_{0}}\frac{\text{1-0.58}\varphi }{\varphi }}$$

where *C*_1_ and *C*_0_ are two constants depending on the network architecture (cubical, tetrahedral or octahedral) whose values are defined by the Scherer theory ([33]; for a cubical network, *C*_0_ = 1 and *C*_1_ = 3$\pi$). The combination of Eq. (18) and Eq. (17) allows expressing the ratio *D*/*D*_0_ as function of the polymer volume fraction (φ) or the average mesh size ($\xi$_a_):

19 $$\frac{\text{D}}{{\text{D}}_{0}}= (1-\frac{{2}{\text{r}}_{\text{s}}/{\text{r}}_{\text{f}}}{\sqrt{\frac{{\text{C}}_{1}}{{\text{C}}_{0}} \, \frac{1-0.58\varphi }{\varphi }}}){\text{e}}^{-{\text{Y}}(\frac{\varphi }{1-\varphi })}$$

20 $$\frac{\text{D}}{{\text{D}}_{0}}=(1-\frac{2}{{\xi }_{\text{a}}^{+}}){\text{e}}^{-\left(\frac{\text{Y}}{{({\xi }_{\text{a}}^{+} \, \frac{{\text{r}}_{\text{s}}}{{\text{r}}_{\text{f}}})}^{2}\frac{{\text{C}}_{0}}{{\text{C}}_{1}}-0.42}\right)}\qquad\quad{\xi }_{\text{a}}^{+}= \frac{{\xi }_{\text{a}} \, }{{\text{r}}_{\text{s}}}$$

Figure 12, Eq. (18) shows Eqs. (19) and (20) trend assuming different values of the ratio *r*_s_/*r*_f_.

The advantage connected with the use of these equations consists in the possibility of simultaneously accounting for both the effect of volume fraction and average mesh size on drug diffusivity, an aspect, usually not explicitly dealt with in literature.

## Appendix 2

**Table 1:** Shear modulus (*G*), average mesh size ($\xi$_a_), average value of the inverse relaxation time ((1/*T*_2_)_m_), and percentage (*A*_i%_) of the different relaxation times *T*_2i_ and mesh size $\xi$_i_

Sample	*G*(Pa)	$\xi$_a_ (nm)	(1/*T*_2_)_m_ ms^−1^	*A*_i%_	*T*_2i_(ms)	$\xi$_i_(nm)
*Before chest physiotherapy*						
**1**	4.5	120	1.767 × 10^−3^	17.5	2182	961
				42.8	798	183
				39.7	344	68
**2**	7	100	4.079 × 10^−3^	19.4	612	280
				65.8	261	107
				14.8	119	47
**3**	7.2	103	1.278 × 10^−3^	43.6	2095	501
				41.3	775	102
				15.1	281	31
**4**	2.7	142	1.379 × 10^−3^	34.5	1463	429
				43.9	730	161
				21.6	292	56
**5**	18	76	4.360 × 10^−3^	6.9	955	400
				42.6	365	125
				50.5	162	52
**6**	15	83	2.910 × 10^−3^	31.5	511	130
				68.5	299	71
**7**	58	51	5.200 × 10^−3^	56.9	261	71
				43.1	143	38
**8**	21	72	1.952 × 10^−3^	32.9	1218	222
				51.6	575	83
				15.5	197	25
**9**	13	84	3.219 × 10^−3^	24.2	1047	362
				42.6	435	122
				33.2	165	43
**10**	10	92	6.645 × 10^−3^	21.5	330	212
				78.5	131	79
**11**	30	64	7.014 × 10^−3^	20.4	355	169
				79.6	124	55
**12**	3	135	0.985 × 10^−3^	53.7	2780	1089
				29.4	989	131
				16.9	341	36
**13**	34	61	2.523 × 10^−3^	22.7	866	155
				71.0	389	60
				6.3	145	21
**14**	2	153	1.994 × 10^−3^	16.7	1831	956
				45.3	861	296
				32.3	364	106
				5.7	117	32
**15**	19	74	3.434 × 10^−3^	40.7	502	136
				50.9	270	68
				8.4	115	28
**16**	28	65	1.692 × 10^−3^	39.4	935	116
				60.6	476	51
**17**	14	83	2.486 × 10^−3^	10.5	2033	830
				62.0	571	124
				27.5	204	40
**18**	35	61	4.729 × 10^−3^	8.4	632	206
				70.2	248	72
				21.4	121	34
**19**	17	77	6.794 × 10^−3^	13.1	353	197
				52.7	133	70
				34.2	139	73
**20**	2	152	2.936 × 10^−3^	18.4	680	338
				62.6	384	174
				19.0	183	78
**21**	10	94	3.462 × 10^−3^	21.1	875	345
				55.3	343	114
				23.6	147	46
*After chest physiotherapy*						
**1**	6	108	1.494 × 10^−3^	15.0	2191	716
				52.8	828	142
				32.2	408	61
**2**	3.7	100	2.926 × 10^−3^	20.5	1108	541
				44.6	441	171
				34.9	202	73
**3**	4.3	122	0.518 × 10^−3^	86.0	2322	189
				14.0	946	38
**4**	4.2	142	1.708 × 10^−3^	38.3	1463	429
				32.4	730	161
				29.3	292	56
**5**	49.5	54	4.576 × 10^−3^	14.1	1224	428
				22.5	483	130
				45.4	205	51
				18.0	101	24
**6**	11	90	1.371 × 10^−3^	30.5	1632	289
				60.2	699	85
				9.3	287	31
**7**	19.6	74	3.628 × 10^−3^	23.5	500	143
				66.7	288	77
				9.8	116	30
**8**	2.6	144	2.435 × 10^−3^	20.0	1117	498
				61.0	492	177
				19.0	187	61
**9**	5.7	84	1.657 × 10^−3^	36.4	1924	618
				33.8	812	160
				29.8	283	47
**10**	3.2	135	0.763 × 10^−3^	57.2	2067	312
				36.6	1152	111
				6.2	368	27
**11**	11	89	3.903 × 10^−3^	21.5	1176	557
				37.6	352	126
				40.9	154	52
**12**	0.1	450	1.311 × 10^−3^	36.1	2439	3364
				35.7	1194	827
				21.9	487	263
				6.3	152	74
**13**	8.4	98	2.210 × 10^−3^	46.2	628	143
				53.8	365	77
**14**	9.7	93	2.425 × 10^−3^	14.7	979	267
				67.5	477	110
				17.8	207	44
**15**	36.8	60	2.197 × 10^−3^	18.0	1540	304
				47.8	609	84
				34.2	264	33
**16**	2.3	151	1.399 × 10^−3^	9.5	1797	597
				45.4	968	224
				45.1	514	102
**17**	0.7	223	0.554 × 10^−3^	72.6	2296	384
				27.4	1153	106
**18**	9.3	94	3.288 × 10^−3^	66.0	342	107
				17.7	667	232
				16.3	149	44
**19**	2.7	142	2.818 × 10^–3^	42.8	581	249
				57.2	275	107
**20**	4.9	117	2.027 × 10^−3^	73.8	627	155
				26.2	308	69.0
**21**	10.4	92	2.155 × 10^−3^	32.5	1386	386
				46.0	509	103
				21.5	211	39
